# Cellular senescence in acute human infectious disease: a systematic review

**DOI:** 10.3389/fragi.2024.1500741

**Published:** 2024-11-15

**Authors:** William C. Miller, Stephanie Wallace, William Kamm, Erin Reardon, Nicole Theis-Mahon, Matthew J. Yousefzadeh, Elizabeth L. Schmidt, Laura J. Niedernhofer, Michael A. Puskarich

**Affiliations:** ^1^ University of Minnesota Medical School, Minneapolis, MN, United States; ^2^ Department of Emergency Medicine, University of Minnesota and Hennepin Healthcare, Minneapolis, MN, United States; ^3^ Woodruff Health Sciences Center Library, Emory University, Atlanta, GA, United States; ^4^ Health Sciences Library, University of Minnesota, Minneapolis, MN, United States; ^5^ Department of Medicine, Columbia Center for Healthy Longevity, Columbia Center for Translation Immunology, Columbia University Medical Center, New York, NY, United States; ^6^ Institute on the Biology of Aging and Metabolism, University of Minnesota, Minneapolis, MN, United States

**Keywords:** cellular senescence, aging, infectious disease, frailty, systematic review

## Abstract

**Introduction:**

*Acute infectious disease represents a significant cause of mortality and morbidity in elderly individuals admitted to the hospital. In its extreme, it presents as sepsis, a systematic inflammatory and immunologic response responsible for self-injurious organ injury. As individuals age, a unique set of factors including immunosenescence predispose them to acquiring an infection and a worse clinical prognosis.* This systematic review explores the relationship between cellular senescence, an age-related inflammatory phenomenon, with acute human infectious disease.

**Methods:**

Embase via OVID, Scopus, Web of Science, Global Index Medicus, Cochrane Library via Wiley, and ClinicalTrials.gov were queried. Included studies must have compared at least one of the following measures of cellular senescence between patients with an infection and without an infection: cell cycle inhibition measured via levels of *p16* ^*INK4a*^ and/or *p21* ^*CIP1*^, short telomere length, DNA damage via ɣH2AX, high senescence-associated β galactosidase activity, and inflammation via the detection of senescence associated secretory phenotype (SASP). Manuscripts were screened and data collected via two independent reviewers.

**Results:**

A total of 15,828 studies were screened after duplicates were removed. One hundred and fifty-three full-text articles were assessed for eligibility and a total of 16 original articles were included in analysis. Of the 16 original articles included, 12 (75%) articles were centered on SARS-CoV-2, 2 (12.5%) articles utilized patients infected with *Leishmania braziliensis*, 1 (6.25%) with *Plasmodium falciparum*, and 1 (6.25%) with Hepatitis C.

**Conclusion:**

Current literature demonstrates robust upregulation of markers of cellular senescence in the setting of acute SARS-CoV-2, *P. falciparum*, *L. braziliensis*, and hepatitis C virus, and that markers of senescence correlate with disease severity and persist for months after resolution. Limitations in the number and types of infectious organisms studied, low sample sizes, modest longitudinal sampling, and a lack of consistency in markers measured, the method of measurement, and the definition of normal values represent ongoing gaps in the literature.

**Systematic Review Registration:**

https://www.crd.york.ac.uk/prospero/display_record.php?RecordID=421473, Identifier CRD42023421473.

## Introduction

Acute infectious diseases represent a leading international cause of death, and in its most severe manifestation presents as sepsis. Sepsis is a life-threatening condition with organ dysfunction due to a dysregulated host response to an infection, as defined by the Third International Consensus Definitions for Sepsis and Septic Shock (Singer et al., 2016). It affects approximately 48 million adults per year worldwide, with 11 million sepsis-related deaths reported (Rudd et al., 2020). The pathophysiology of sepsis is a complex spatiotemporal connected pathway of innate and adaptive immune system dysfunction leading to an early pro-inflammatory response and eventual immunosuppression leading to end-organ failure (Jarczak et al., 2021). Despite advances in treatment, sepsis-related hospital mortality continues to approach 50% and represents a significant burden on healthcare spending (Markwart et al., 2020; van den Berg et al., 2022). The underlying etiology of a sepsis is broad and includes bacterial, viral, and fungal acute infectious pathogens (Koçak Tufan et al., 2021; Lin et al., 2018).

Uniquely, elderly individuals pose a particularly high risk of developing sepsis as a response to routine acute infectious disease with increased mortality and morbidity (Esme et al., 2019; Guidet et al., 2018; Yoshikawa and Norman, 2017), and make up the majority of individuals hospitalized (Rowe and McKoy, 2017). It has s been estimated that individuals > 60 years old demonstrate a 20% increased risk of developing sepsis (Martin et al., 2006). As individuals age, cognitive and physical domains diminish in capacity, leading to a state of frailty, predisposing individuals to infections and deleterious downstream morbidity and mortality (Trevisan et al., 2023). A commonly described driver of human aging that has been described across disease states is the phenomenon of cellular senescence, which may contribute to adverse outcomes in the setting of acute infectious diseases and the subsequent development of sepsis (Nasa et al., 2012).

Cellular senescence is characterized by a stable exit from the cell cycle with continued secretion of a milieu of pro-inflammatory cytokines and chemokines that, through a paracrine mechanism, induce further cellular senescence of nearby cells (Gorgoulis et al., 2019). The senescence associated secretory phenotype (SASP) also drives a state of chronic sterile inflammation that when coupled with an infection causes a propensity for cytokine storm and organ damage (Camell et al., 2021). Characteristically, senescent cells are particularly difficult for the immune system to clear, thus resulting in an accumulation of senescent cells as individuals age (Prata nd Mckoy, 2018). Beyond local senescent cell accumulation and burden are the deleterious effects of age on an individual’s immune function, dubbed immunosenescence, resulting in an increased predilection for infection and decreased or altered response to infection (Fulop et al., 2018; Camell et al., 2021).

Senescent cells can be selectively targeted with a new class of drugs “senotherapeutics” which consists of two classes (Zhang et al., 2023). Senolytics selectively kill senesecent cells, while senomorphics modulate their proinflammatory secretions (Zhang et al., 2023). The potential role of cellular senescence and the role of senolytic medications to reduce their burden in the setting of acute infectious diseases has been demonstrated in preclinical models. However, the data in humans is less robust. Given the critical role of the immune system in the pathophysiology of sepsis, there is also a growing understanding that acute infections can increase the senescent cell burden, and how that negatively impacts clinical recovery. Despite this evolving knowledge based, there remains a substantial gap in the literature studying cellular senescence in sepsis. Previous work has sought to describe the mechanistic involvement of senescence in acute infection using *in-vitro* models and suggest a critical role of this effect of aging on sepsis pathophysiology, however a paucity of data in human patients exists (Reyes et al., 2023; Schmitt et al., 2023; Kelley et al., 2020). To address this gap, t he goal of this systematic review is to comprehensively summarize the current state of medical literature as it relates to cellular senescence in acute human infections, excluding *in-vitro* models, to serve as a basis for future identifying literature gaps to inform explorations in more severe forms of infections, such as sepsis, more specifically.

## Materials and methods

The present systematic review was performed in compliance with the PRISMA 2020 (Preferred Reporting Items for Systematic Reviews and Meta-Analyses) guidelines (Open Access Page et al., 2021) and the study protocol was registered using the International Prospective Register of Systematic Reviews, CRD42023421473. A PRISMA 2020 checklist is available via Supplementary Table S1.

### Search strategy

The search strategy to identify relevant articles was built by a health sciences librarian and tested for sensitivity in Ovid MEDLINE using medical subject (MeSH) headings, keywords, and synonyms to encompass the concepts of cell senescence and infection. The search was then translated to an additional six databases: Embase via OVID, Scopus, Web of Science, Global Index Medicus, Cochrane Library via Wiley, and ClinicalTrials.gov. Searches were run from the inception of each database through 8 August 2022 and an updated search was run on 13 December 2023. No limitations or search filters were applied. The full Ovid Medline (R) All search strategy can be found in the appendix. A broad search strategy was employed so as to not miss articles with useful data that included, but were not exclusive of, human patients rather than *in-vitro* models.

### Study selection

Search results were imported to Covidence for automatic deduplication and screening (Covidence systematic review software, 2024). Two investigators (WM and SW) independently reviewed the titles and abstracts of all studies. In cases of disagreement, conflict was resolved by discussion between the two investigators with the help of a third investigator (MP) if needed. Disagreement on abstract screening was resolved prior to accessing the full article. The same two investigators independently reviewed the full text of included studies in Covidence with the same process of conflict resolution.

We included studies reporting original data comparing levels of cellular senescence in human patients diagnosed with an acute infection compared to patients without an infection. Studies exclusively reporting data on animals or plants, including animal cell lines, were excluded. If a study presented data about human patients and animals or plants, including cell lines, it was included for full text review to collect only the data pertaining to human patients. If this was not possible, the study was excluded from full text review. The initial search strategy was made intentionally broad to ensure that articles focused on *in vitro* results, but that included patient biospecimens as an ancillary portion of the manuscript were captured. Reviews discussing the effects of cellular senescence and infection were also included for full text review, where references were searched for any studies reporting original data that fit the inclusion criteria.

Study inclusion criteria were determined prior to literature search and author review/screening. For a study to be included, it must have compared at least one of the following measures of cellular senescence between patients with an infection and without an infection: cell cycle inhibition measured via levels of *p16* ^*INK4a*^ and/or *p21* ^*CIP1*^, short telomere length, DNA damage via ɣH2AX, high senescence-associated β galactosidase activity, and inflammation via the detection of SASP. SASP consists not only of pro-inflammatory cytokines, chemokines, but also a heterogeneous mix of growth factors and matrix remodeling enzymes (Coppé et al., 2010). The strength of study was based on the number of senescent cell biomarkers that were measured as none of them are specific to senescent cells. If a study reported telomere length data, it must have also reported another measure of cellular senescence due to the low specificity of telomere length and cellular senescence (Victorelli and Passos, 2017). In this systematic review, patients must have been in the acute phase of infection, confirmed by a diagnostic test. Chronic infections including but not limited to HIV, hepatitis B and C, and chronic cytomegalovirus were excluded.

The primary outcome measure was the difference in markers of cellular senescence between acutely infected patients and controls. Additional secondary outcome measures included but not limited to the correlation of senescence levels with disease severity, mortality, and healthcare resource utilization, as available.

### Data extraction

One investigator independently extracted data while the second investigator verified data for accuracy and completeness. Data collected included the type of infection studied, how the infection was diagnosed or verified, senescence markers studied, and the levels of senescence measured in patients with acute infection and without. Because numerical values of senescence markers were inconsistently reported across studies, descriptive analyses were extracted. Other variables collected included study type, publication year, and study limitations.

### Statistical analysis

A meta-analysis combining the extracted data to determine overall cellular senescence with each infectious agent could not be performed due to a lack of standardized senescent marker identification (Ogrodnik et al., 2024). Inter-rater reliability assessed at both title/abstract review and full-text review stages using Cohen’s kappa calculated by Covidence (Covidence systematic review software, 2024).

## Results

### Selection of included studies

**FIGURE 1:**
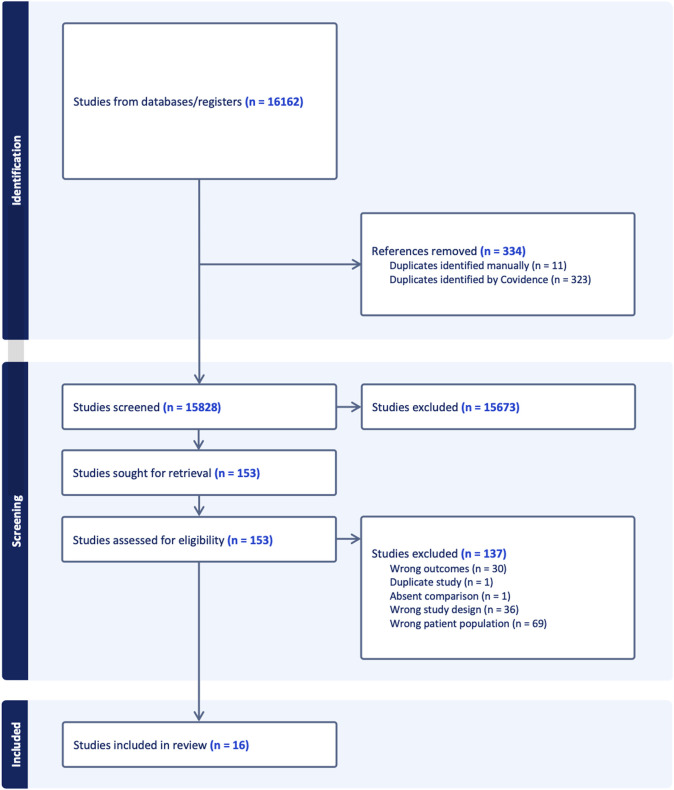
Study flow diagram for identified, screened, and excluded articles.

Figure 1 provides a detailed flow diagram of study screening and selection. A total of 15,828 studies were screened after duplicates were removed, reflecting the comprehensive search strategy, but emphasizing the observation that most data in the field is based in preclinical and/or *in vitro* models. One hundred and fifty-three full-text articles were assessed for eligibility and a total of 16 original articles were included in analysis. The inter-rater reliability was calculated using Cohen’s kappa. For the title/abstract screening it was 0.38 and for the full-text screening it was 0.66.

### Included study characteristics

Of the 16 original articles (Table 1) included after screening, 12 (75%) articles were centered on SARS-CoV-2 as the primary disease (Froidure et al., 2020; Zheng et al., 2020; Evangelou et al., 2021; 2022; Lee et al., 2021; Wang et al., 2021; 2023; Lekva et al., 2022; Lipskaia et al., 2022; Nguyen et al., 2022; Roh et al., 2022; Lin et al., 2023). Two (12.5%) articles utilized patients infected with *Leishmania braziliensis*, 1 (6.25%) with *Plasmodium falciparum*, and 1 (6.25%) with Hepatitis C (Asghar et al., 2018; Covre et al., 2019; Fantecelle et al., 2021; Martín-Escolano et al., 2023). A total of 1239 patients were included across all studies. Of those patients, 443 (35.8%) patients had a diagnosis of an acute infection and 710 (57.3%) were included as controls. The remaining 86 (6.9%) individuals were not identified as infected or a control specifically. Generally, controls were defined as individuals with similar comorbidities without acute infection. A total of 11 (68.8%) studies clearly defined age-matching controls to experimental groups. The gender distribution was provided in 13 (81.25%) articles, and average/median age was provided in 13 (81.25%) articles (Table 1). Studies typically reported a difference in measured senescence markers between infected patients and controls. In a subset of included studies, the severity of disease or relative disease load was also reported (Table 1).

**TABLE 1:** Summary of studies included in the analysis with their infectious disease of interest, number of subjects and controls, and measured senescence markers.

Study	Pathogen	Participants (n)	Healthy controls (n)	Senescence markers
Evangelou et al. (2022) ^b,c^	SARS-CoV-2	11	43	p16^INK4a^, ɣH2AX, SASP, SenTraGor™
Froidure et al. (2020) ^b^	SARS-CoV-2	70	491	SA-β-gal, Telomere length
Lee et al. (2021) ^b^	SARS-CoV-2	24	5	p16^INK4a^, p21^CIP1^, H3K9me3, SASP, Lipofuscin
Lekva et al. (2022) ^b,c^	SARS-CoV-2	97	22	p16^INK4a^, p21^CIP1^, SA-β-gal, Telomerase activity
Lin et al. (2023) ^b,c^	SARS-CoV-2	24	12	p16^INK4a^, p21^CIP1^, SASP
Lipskaia et al., 2022 ^b,c^	SARS-CoV-2	9	2	p16^INK4a^, p21^CIP1^, SASP
Nguyen et al. (2022) ^b^ ^,^ ^c^	SARS-CoV-2	28	8	SASP
Evangelou et al. (2021) ^c^	SARS-CoV-2	10	10	P16, SASP, SenTraGor™
Roh et al. (2022) ^b,c^	SARS-CoV-2	54	26	SASP
Wang et al. (2021) ^c^	SARS-CoV-2	5	4	p16^INK4a^, p21^CIP1^, p53, SASP
Wang et al. (2023) ^b^	SARS-CoV-2	3	3	mTOR, MAPK, p53 pathways
Zheng et al. (2020) ^b^ ^,^ ^c^	SARS-CoV-2	Cohort 1: 56^a^ Cohort 2: 8 Cohort 3: 22		SASP
Fantecelle et al. (2021) ^b^	*Leishmania Braziliensis*	21	7	p16^INK4a^, p21^CIP1^, p38, ATM, SASP
Covre et al. (2019) ^b,c^	*Leishmania Braziliensis*	17	15	ɣH2AX, SASP, Telomere length
Asghar et al. (2018) ^b,c^	*Plasmodium Falciparum*	38	38	p16^INK4a^, Telomere length, Telomerase activity
Martin-Escolano et al. (2023) ^b,c^	Hepatitis C	32	24	SASP, Immune checkpoint biomarkers

^a^ Cohort one consisted of young healthy adults (20–45 years old) and aged healthy adults (≥60 years old); Cohort 2 consisted of young health adults (30–45 years old), aged healthy adults (≥60 years old), young SARS-CoV-2 (30–50 years old), and aged SARS-CoV-2 (≥70 years old); Cohort 3 consisted of young health adults (30–45 years old), aged healthy adults (≥60 years old), young recovered SARS-CoV-2 (30–50 years old), and aged recovered SARS-CoV-2 (≥70 years old).

^b^ Denotes studies clarifying gender distribution.

^c^ Deontes studies clarifying age-matched controls.

Abbreviations: p16^INK4a^ (cyclin dependent kinase inhibitor 2A); p21^CIP1^ (cyclin dependent kinase inhibitor 1); SASP (Senescence associated secretory phenotype); SenTraGor™ (Antibody enhanced detection of senescent cells); yH2AX (gamma H2A histone family member X), SA-β−gal (Senescence associated beta-galactosidase activity); H3K9me3 (Histone H3 Lysine 9 trimethylation); GDF15 (Growth differentiation factor 15); F3 (Coagulation factor III); mTOR (mammalian target of rapamycin); MAPK (mitogen activated protein kinase); p53 (Tumor protein P53); p38 (mitogen activated protein kinase 14); ATM (ataxia telangiectasia mutated).

### Viral etiologies

#### SARS-CoV-2

The majority of included articles analyzed markers of cellular senescence in SARS-CoV-2 or COVID-19 (n = 12). A total of 9 (75%) COVID-19 articles measured cell cycle inhibitors including p16, p21, and p53. Of the 12 articles, 9 (75%) included some measure of senescence associated secretory phenotype (SASP), 3 (25%) included a quantification of lipofuscin, and 2 (16.7%) measured telomere length/telomerase activity. A total of 12 (100%) of COVID-19 articles found an association between acute COVID-19 infection and an increased burden of cellular senescence (Table 2).

**TABLE 2:** Summary of findings from studies included in the analysis specifically investigating SARS-CoV-2.

Study	Senescence markers	Sample type	Primary findings
Evangelou et al. (2022)	p16, yH2AX, SASP, SenTraGor	AT2 Lung Cells	↑p16, ↑SenTraGor positivity, and ↑SASP (*p* < 0.0001)
Froidure et al. (2020)	SA-β−gal, Telomere length	Leukocytes	Shorter telomeres than controls (*p* < 0.001) and ↑SA- β−gal positivity
Lee et al. (2021)	p16, p21, H3K9me3, SASP, Lipofuscin	Airway Mucosa; macrophages	↑p16, ↑p21, ↑H3K9me3, ↑ lipofuscin, and ↑IL8
Lekva et al. (2022)	p16, p21, SA- β−gal, telomere-associated SASP	Plasma	↑p16 and telomere-associated SASP but normal p21 and SA- β−gal 3 months after hospitalization (*p* < 0.05)
Lin et al. (2023)	p16, p21, SASP	PBMC	p21 (*p* < 0.05) correlated with disease severity and SenMayo SASP gene-set was upregulated
Lipskaia et al. (2022)	p16, p21, SASP, GDF15	Epithelial ciliated; club cells	↑p16, ↑p21, ↑uPAR, ↑CXCL8, ↑IGFBP3, and ↑GDF15 (*p* < 0.0001)
Nguyen et al. (2022)	SASP, F3	Macrophages; Epithelial cells	Macrophages had moderate levels of SASP. Epithelial cells displayed ↑SASP in SARS-CoV-2 patients with increased expression of F3 in severe cases (*p* < 0.05)
Evangelou et al. (2021)	p16, SASP, SenTraGor	AT2 Lung Cells	Greater reactivity to SenTraGor, ↑p16 immunostaining, and co-expression of IL-1β and IL-6
Roh et al. (2022)	SASP	Plasma	↑SASP expression with cardiac involvement of SARS-CoV-2 (*p* < 0.05)
Wang et al. (2021)	p16, p21, p53, SASP	Lung Tissue	↑p16, ↑p21, ↑IL-6, ↑p53, and ↑SASP
Wang et al. (2023)	mTOR, MAPK, p53 pathways	Testicular Tissue	Senescence mediated by MAPK (*r* = 0.999, *p* < 0.001), mTOR, and p53 signaling is positively correlated with SARS-CoV-2 disease in testes
Zheng et al. (2020)	SASP	Peripheral T-Cells; monocytes	↑SASP hallmark genes (*CDKN* family)

#### Hepatitis C

Martín-Escolano et al. described the correlation of acute Hepatitis C Virus (HCV) infection and increased senescent cell burden (Table 3). The SASP and immune checkpoint signaling molecules were correlated to spontaneously cleared HCV infection compared to individuals without evidence of infection. They demonstrated an increase in 13 immune checkpoint signaling molecules and 13 SASP proteins in the HCV spontaneous clearance group, measured approximately 2 years post-infection clearance.

**TABLE 3:** Summary of findings from studies included in the analysis investigating *Leishmania braziliensis*, *Plasmodium falciparum*, or hepatitis C.

Study	Disease	Senescence markers	Sample type	Primary findings
Asghar et al. (2018)	*P. falciparum*	p16, Telomere length, Telomerase activity	Whole Blood	↑p16, ↓telomerase activity, and telomere shortening (*p* < 0.05). Over time telomerase activity increases and telomere length is gradually restored
Covre et al. (2019)	*L. braziliensis*	γH2AX, Telomere length, Telomerase activity, CD57	CD8 T Cells	↑expression of SASP markers including CD57, KLRG1, p38, and γH2AX (*p* < 0.001), ↓telomeres (*p* < 0.0001) and ↓telomerase expression (*p* < 0.001)
Fantecelle et al. (2021)	*L. braziliensis*	p16, p21, p38, ATM, Sestrin 2	Skin	↑p16, p21, p38, ATM, and Sestrin 2 (*p* < 0.0001); positively correlated with lesion size and parasitic load and independent of patient age
Martín-Escolano et al. (2023)	Hepatitis C	SASP, immune checkpoint biomarkers	Plasma	↑SASP and immune checkpoint biomarkers more than 2 years after infection (*p* < 0.05)

### Protozoal etiologies

#### Leishmania Braziliensis

Of the 16 total included studies, 2 (12.5%) analyzed acute *Leishmania Braziliensis* infection (Table 3). Covre et al. correlated telomere length, SASP, and DNA damage marker ɣH2AX to acute infection. They detailed the accumulation of senescent circulating T cells with homing to the skin associated via increased circulating SASP in the acute infectious phase. Fantacelle et al. analyzed the cell cycle inhibitors p16^INK4a^ and p21^CIP1^, the MAP kinase-activating p38, and SASP. This work built upon the aforementioned study by Covre et al., detailing accumulating senescent burden in CD8^+^ effector memory, T_EMRA_ (terminal effector memory T cells), and NK cells localized to cutaneous infection.

#### Plasmodium falciparum

The article by Asghar et al. analyzed acute *P. falciparum* infection and cellular senescence (Table 3). This longitudinal study specifically measured the expression of the cell cycle inhibitor p16^INK4a^, telomerase activity, and telomere length in infected individuals in the acute infectious phase and 12 months post infection. There was a demonstrable increase in *p16* ^*INK4a*^ (*CDKN2A)* expression, reduced telomerase activity, and telomere shortening during the acute infectious phase when pairwise compared to the healthy individual at 12-month post infection.

## Discussion

In this systematic review, we identified a relative paucity of human studies examining the effect of acute infectious diseases on cellular senescence. However, in all four of the conditions studied, patients with acute infection demonstrated higher senescent cell markers than controls. Senescence markers were amplified in serum, infected tissue, and peripheral immune cells, including T cells, monocytes, and macrophages (Asghar et al., 2018; Covre et al., 2019; Zheng et al., 2020; Evangelou et al., 2021; Fantecelle et al., 2021; Lee et al., 2021; Wang et al., 2021; Lipskaia et al., 2022; Nguyen et al., 2022; Roh et al., 2022; Martín-Escolano et al., 2023). A large number of manuscripts were screened for inclusion, however ultimately excluded due to them including exclusively *in-vitro* or cellular models without human patient samples.

There is a strong pool of literature supporting the upregulation of p16^INK4a^ and p21^CIP1^ as reliable senescence markers in infected tissue (Evangelou et al., 2021; 2022; Fantecelle et al., 2021; Lee et al., 2021; Lipskaia et al., 2022). p16^INK4a^ is the most well-studied marker of senescence in the peripheral serum monocytes and has been shown to be increased in acute disease states (Asghar et al., 2018; Zheng et al., 2020; Lekva et al., 2022). Additionally, expression of p16^INK4a^ in peripheral T cells is a viable marker of normal human aging outside of infectious processes and appears to be correlated to patient frailty within and across age demographics (Liu et al., 2009; Englund et al., 2021). In the preclinical literature, this phenomenon does not appear to be exclusive to the four conditions included in this review. Senescence has been observed *in vitro* or *ex vivo* with other acute infections including influenza A virus and respiratory syncytial virus (Li et al., 2017; Schulz et al., 2024) but also in chronic viral infections including CMV, HCV, and HIV (Montano et al., 2022; Raviola et al., 2024). Senescence driven by these chronic viral infections is believed to accelerate cellular aging and contribute to a pro-tumor micro- and macro-environments (Asghar et al., 2018; Froidure et al., 2020).

Mechanistically, it appears that age-related accumulation of senescent cells predisposes an individual to acute infection with a cellular polarization towards inflammatory states. This is true for both peripheral tissue and circulating immune cells. Subsequently, the acute infection stimulates further the accumulation of senescent cells, which exacerbates disease phenotype through a positive feedback loop, ultimately creating excess inflammation (Zheng et al., 2020; Fantecelle et al., 2021; Wang et al., 2021; Lipskaia et al., 2022; Roh et al., 2022). This is supported by senescence induction by upregulation of MAPK signaling in SARS-CoV-2 (Wang et al., 2023) and excessive stem cell replication in acute malaria infection (Asghar et al., 2018). Additionally, SASP secretion in cutaneous *L. braziliensis* appears to create a positive-feedback loop with the immune system which induces substantial inflammation (Fantecelle et al., 2021). Based on the methodology of the reported studies, however, it remains unclear whether patients with increased senescence are more prone to acute infection, whether acute infections in and of themselves increase cellular senescence, or both.

Chronic sequelae of acute infections may also be a consequence of enhanced cellular senescence. In SARS-CoV-2, senescence caused by acute infection is shown to contribute to long-standing changes like emphysema and fibrosis by accelerating age-related changes (Wang et al., 2021). Lipskaia et al. postulated there may be a causal link between epithelial cell senescence and vascular thrombosis (Lipskaia et al., 2022). Subsequent studies revealed upregulated senescence, F3, and von Willebrand factor in epithelial cells of patients with severe SARS-CoV-2 (Wang et al., 2021; Nguyen et al., 2022). Furthermore, the antithrombotic protein ADAMTS13 was shown to be decreased and to have a strong inverse relation with cardiac injuries like acute coronary syndrome (Roh et al., 2022). All of these changes contribute to thrombosis of the pulmonary vasculature, which is a well-known cause of pulmonary fibrosis and may be implicated in the development of post-COVID-19 syndrome.

Following resolution of these acute infections, we see mixed results whether senescence markers persist or return to baseline. Patients infected with *P. falciparum* showed resolution of serum senescence markers to baseline within the year following acute infection (Asghar et al., 2018). Following recovery of acute hepatitis C, however, serum senescence markers persist for over 2 years and are suspected to contribute to T cell exhaustion (Martín-Escolano et al., 2023). Serum samples from patients with severe SARS-CoV-2 showed persistently upregulated SASP 3 months after resolution of infection in an age-dependent and age-independent manner further supporting involvement of senescence in post-COVID-19 syndrome (Lekva et al., 2022).

Importantly, senescence markers may be clinically significant as predictors of disease presence and severity and can be used to guide treatment. The studies included in this review show the burden of senescence markers correlates with disease severity. For SARS-CoV-2, this is true of numerous markers including p21^CIP1^ in peripheral blood mononuclear cells (Lin et al., 2023), telomere shortening in peripheral blood T cells (Froidure et al., 2020), serum SASP profile at time of admission (Roh et al., 2022), and MAPK signaling in the testes (*r* = 0.999, *p* < 0.001) (Wang et al., 2023). *P. falciparum* infection is also shown to be correlated with increased p16^INK4a^ expression, decreased telomerase activity, and shortened telomeres in peripheral white blood cells (Asghar et al., 2018). Finally, in *L. braziliensis* infection, the size of cutaneous lesions and parasitic load correlated with cutaneous SASP marker expression (Fantecelle et al., 2021).

Given the substantial involvement of cellular senescence in acute infection, this process may be a viable drug target. The senolytics navitoclax, fisetin, and quercetin plus dasatinib were shown to be effective at reducing senescent phenotype *in vivo* models of SARS-CoV-2 infection. Moreover, animal models treated with these senolytics displayed meaningful improvement of disease phenotype albeit with moderate adverse effects (Lee et al., 2021). F3 inhibitors are also being explored as possible therapeutic agents to prevent dysregulation of thrombosis seen with epithelial cell senescence in SARS-CoV-2 (Nguyen et al., 2022).

The mechanism by which cellular senescence influences an individual’s risk of acquiring infection and that infection being severe in nature are numerous and dependent on the infectious agent. Generally, it involves an alteration in immune cell populations systemically, a decline in the innate and adaptive immune function, and chronic inflammation (Wrona et al., 2024; Marrella et al., 2022). Although outside the scope of this review, these mechanisms are explored and reported in other narrative and literature reviews and we refer interested readers to the following excellent summaries (Reyes et al., 2023; Wrona et al., 2024; Marrella et al., 2022; Li et al., 2023).

The key limitation of this review was the study populations. Most included studies were limited to populations of severely to critically ill patients which raises concerns regarding the generalizability of these findings (Froidure et al., 2020; Evangelou et al., 2022). Further clinical research is essential to develop a thorough understanding of the role of cellular senescence in acute infection. Mechanistic questions remain including how acute infections affect the rate of senescent cell accumulation and aging and if these are transient events. There are also practical challenges to the clinical use of senescence markers including when they should be measured during a disease course and which markers should be measured. Furthermore, it is unclear if nucleic acid or protein assays are more reliable in clinical populations.

## Conclusion

Current literature demonstrates robust upregulation of markers of cellular senescence in the setting of acute SARS-CoV-2, *P. falciparum*, *L. braziliensis*, and hepatitis C virus, and that markers of senescence correlate with disease severity and persist for months after resolution. Limitations in the number and types of infectious organisms studied, low sample sizes, modest longitudinal sampling, and a lack of consistency in markers measured, the method of measurement, and the definition of normal values represent ongoing gaps in the literature.

## Data Availability

The original contributions presented in the study are included in the article/Supplementary Material, further inquiries can be directed to the corresponding author.

## References

[B1] Asghar M. Yman V. Homann M. V. Sondén K. Hammar U. Hasselquist D. (2018). Cellular aging dynamics after acute malaria infection: a 12-month longitudinal study. Aging Cell 17, e12702. 10.1111/acel.12702 29143441 PMC5771395

[B2] Camell C. D. Yousefzadeh M. J. Zhu Y. Prata L. G. P. L. Huggins M. A. Pierson M. (2021). Senolytics reduce coronavirus-related mortality in old mice. Science 373, eabe4832. 10.1126/science.abe4832 34103349 PMC8607935

[B3] Coppé J.-P. Desprez P.-Y. Krtolica A. Campisi J. (2010). The senescence associated secretory phenotype: the dark side of tumor suppression. Annu. Rev. Pathol. 5, 99–118. 10.1146/annurev-pathol-121808-102144 20078217 PMC4166495

[B4] Covidence systematic review software, Veritas Health Innovation (2024). Melbourne, Australia.

[B5] Covre L. P. Martins R. F. Devine O. P. Chambers E. S. Vukmanovic-Stejic M. Silva J. A. (2019). Circulating senescent T cells are linked to systemic inflammation and lesion size during human cutaneous leishmaniasis. Front. Immunol. 9, 3001. 10.3389/fimmu.2018.03001 30662437 PMC6328442

[B6] Englund D. A. Sakamoto A. E. Fritsche C. M. Heeren A. A. Zhang X. Kotajarvi B. R. (2021). Exercise reduces circulating biomarkers of cellular senescence in humans. Aging Cell 20, e13415. 10.1111/acel.13415 34101960 PMC8282238

[B50] Esme M. Topeli A. Yavuz B. B. Akova M. Li H. (2019). Infections in the elderly critically-ill patients. Front. Med. (Lausanne) 6, 118. 31275937 10.3389/fmed.2019.00118 PMC6593279

[B7] Evangelou K. Veroutis D. Foukas P. G. Paschalaki K. Kittas C. Tzioufas A. G. (2021). Alveolar type II cells harbouring SARS-CoV-2 show senescence with a proinflammatory phenotype. bioRxiv, OA4312. 10.1183/13993003.congress-2021.oa4312

[B8] Evangelou K. Veroutis D. Paschalaki K. Foukas P. G. Lagopati N. Dimitriou M. (2022). Pulmonary infection by SARS-CoV-2 induces senescence accompanied by an inflammatory phenotype in severe COVID-19: possible implications for viral mutagenesis. Eur. Respir. J. 60, 2102951. 10.1183/13993003.02951-2021 35086840 PMC8796696

[B9] Fantecelle C. H. Covre L. P. Garcia de Moura R. Guedes H. L. de M. Amorim C. F. Scott P. (2021). Transcriptomic landscape of skin lesions in cutaneous leishmaniasis reveals a strong CD8^+^ T cell immunosenescence signature linked to immunopathology. Immunology 164, 754–765. 10.1111/imm.13410 34432883 PMC8561102

[B10] Froidure A. Mahieu M. Hoton D. Laterre P.-F. Yombi J. C. Koenig S. (2020). Short telomeres increase the risk of severe COVID-19. Aging (Albany NY) 12, 19911–19922. 10.18632/aging.104097 33104521 PMC7655194

[B11] Fulop T. Larbi A. Dupuis G. Le Page A. Frost E. H. Cohen A. A. (2018). Immunosenescence and inflamm-aging as two sides of the same coin: friends or foes? Front. Immunol. 8, 1960. 10.3389/fimmu.2017.01960 29375577 PMC5767595

[B12] Gorgoulis V. Adams P. D. Alimonti A. Bennett D. C. Bischof O. Bishop C. (2019). Cellular senescence: defining a path forward. Cell 179, 813–827. 10.1016/j.cell.2019.10.005 31675495

[B41] Guidet B. Vallet H. Boddaert J. de Lange D. W. Morandi A. Leblanc G. (2018). Caring for the critically ill patients over 80: a narrative review. Ann. Intensive Care 8, 114. 30478708 10.1186/s13613-018-0458-7 PMC6261095

[B13] Jarczak D. Kluge S. Nierhaus A. (2021). Sepsis—pathophysiology and therapeutic concepts. Front. Med. (Lausanne) 8, 628302. 10.3389/fmed.2021.628302 34055825 PMC8160230

[B44] Kelley W. J. Zemans R. L. Goldstein D. R. (2020). Cellular senescence: friend or foe to respiratory viral infections?. Eur. Respir. J. 56, 2002708. 33033152 10.1183/13993003.02708-2020 PMC7758538

[B49] Koçak Tufan Z. Kayaaslan B. Mer M. (2021). COVID-19 and sepsis. Turk. J. Med. Sci. 51, 3301–3311. 34590796 10.3906/sag-2108-239 PMC8771020

[B14] Lee S. Yu Y. Trimpert J. Benthani F. Mairhofer M. Richter-Pechanska P. (2021). Virus-induced senescence is a driver and therapeutic target in COVID-19. Nature 599, 283–289. 10.1038/s41586-021-03995-1 34517409

[B15] Lekva T. Ueland T. Halvorsen B. Murphy S. L. Dyrhol-Riise A. M. Tveita A. (2022). Markers of cellular senescence is associated with persistent pulmonary pathology after COVID-19 infection. Infect. Dis. (Lond.) 54, 918–923. 10.1080/23744235.2022.2113135 35984738

[B16] Li B. Hou D. Guo H. Zhou H. Zhang S. Xu X. (2017). Resveratrol sequentially induces replication and oxidative stresses to drive p53-CXCR2 mediated cellular senescence in cancer cells. Sci. Rep. 7, 208. 10.1038/s41598-017-00315-4 28303009 PMC5428242

[B48] Li Z. Tian M. Wang G. Cui X. Ma J. Liu S. (2023). Senotherapeutics: an emerging approach to the treatment of viral infectious diseases in the elderly. Front. Cell. Infect. Microbiol. 13, 1098712. 37065192 10.3389/fcimb.2023.1098712 PMC10094634

[B40] Lin G.-L. McGinley J. P. Drysdale S. B. Pollard A. J. (2018). Epidemiology and immune pathogenesis of viral sepsis. Front. Immunol. 9, 2147. 30319615 10.3389/fimmu.2018.02147 PMC6170629

[B17] Lin Y. Postma D. F. Steeneken L. S. Melo dos Santos L. S. Kirkland J. L. Espindola-Netto J. M. (2023). Circulating monocytes expressing senescence‐associated features are enriched in COVID‐19 patients with severe disease. Aging Cell 22, e14011. 10.1111/acel.14011 37969056 PMC10726854

[B18] Lipskaia L. Maisonnasse P. Fouillade C. Sencio V. Pascal Q. Flaman J.-M. (2022). Evidence that SARS-CoV-2 induces lung cell senescence: potential impact on COVID-19 lung disease. Am. J. Respir. Cell Mol. Biol. 66, 107–111. 10.1165/rcmb.2021-0205LE 34648725 PMC8803362

[B19] Liu Y. Sanoff H. K. Cho H. Burd C. E. Torrice C. Ibrahim J. G. (2009). Expression of p16(INK4a) in peripheral blood T-cells is a biomarker of human aging. Aging Cell 8, 439–448. 10.1111/j.1474-9726.2009.00489.x 19485966 PMC2752333

[B20] Markwart R. Saito H. Harder T. Tomczyk S. Cassini A. Fleischmann-Struzek C. (2020). Epidemiology and burden of sepsis acquired in hospitals and intensive care units: a systematic review and meta-analysis. Intensive Care Med. 46, 1536–1551. 10.1007/s00134-020-06106-2 32591853 PMC7381455

[B46] Marrella V. Facoetti A. Cassani B. (2022). Cellular senescence in immunity against infections. Int. J. Mol. Sci. 23, 11845. 36233146 10.3390/ijms231911845 PMC9570409

[B21] Martín-Escolano R. Vidal-Alcántara E. J. Crespo J. Ryan P. Real L. M. Lazo-Álvarez J. I. (2023). Immunological and senescence biomarker profiles in patients after spontaneous clearance of hepatitis C virus: gender implications for long-term health risk. Immun. Ageing 20. 10.1186/s12979-023-00387-z PMC10655350 37978401

[B51] Martin G. S. Mannino D. M. Moss M. (2006). The effect of age on the development and outcome of adult sepsis. Crit. Care Med. 34, 15–21. 16374151 10.1097/01.ccm.0000194535.82812.ba

[B22] Montano M. Oursler K. K. Xu K. Sun Y. V. Marconi V. C. (2022). Biological ageing with HIV infection: evaluating the geroscience hypothesis. Lancet Healthy Longev. 3, e194–e205. 10.1016/s2666-7568(21)00278-6 36092375 PMC9454292

[B52] Nasa P. Juneja D. Singh O. Xie L. (2012). Severe sepsis and septic shock in the elderly: an overview. World J. Crit. Care Med. 1, 23–30. 24701398 10.5492/wjccm.v1.i1.23 PMC3956061

[B23] Nguyen D. Jeon H.-M. Lee J. (2022). Tissue factor links inflammation, thrombosis, and senescence in COVID-19. Sci. Rep. 12, 19842. 10.1038/s41598-022-23950-y 36400883 PMC9673213

[B24] Ogrodnik M. Carlos Acosta J. Adams P. D. d’Adda di Fagagna F. Baker D. J. Bishop C. L. (2024). Guidelines for minimal information on cellular senescence experimentation *in vivo* . Cell 187, 4150–4175. 10.1016/j.cell.2024.05.059 39121846 PMC11790242

[B25] Open Access Page M. J. Mckenzie J. E. Bossuyt P. M. Boutron I. Hoffmann T. C. Mulrow C. D. (2021). The PRISMA 2020 statement: an updated guideline for reporting systematic reviews. Syst. Rev. 10, 89. 10.1186/s13643-021-01626-4 33781348 PMC8008539

[B26] Prata L. G. P. L. Ovsyannikova I. G. Tchkonia T. Kirkland J. L. (2018). Senescent cell clearance by the immune system: emerging therapeutic opportunities. Semin. Immunol. 40, 101275. 10.1016/j.smim.2019.04.003 31088710 PMC7061456

[B27] Raviola S. Griffante G. Iannucci A. Chandel S. Lo Cigno I. Lacarbonara D. (2024). Human cytomegalovirus infection triggers a paracrine senescence loop in renal epithelial cells. Commun. Biol. 7, 292. 10.1038/s42003-024-05957-5 38459109 PMC10924099

[B47] Reyes A. Ortiz G. Duarte L. F. Fernández C. Hernández-Armengol R. Palacios P. A. (2024). Contribution of viral and bacterial infections to senescence and immunosenescence. Front. Cell. Infect. Microbiol. 13, 1229098. 10.3389/fcimb.2023.1229098 PMC10518457 37753486

[B28] Roh J. D. Kitchen R. R. Guseh J. S. McNeill J. N. Aid M. Martinot A. J. (2022). Plasma proteomics of COVID-19-associated cardiovascular complications: implications for pathophysiology and therapeutics. JACC Basic Transl. Sci. 7, 425–441. 10.1016/j.jacbts.2022.01.013 35530264 PMC9067411

[B29] Rowe T. A. McKoy J. M. (2017). Sepsis in older adults. Infect. Dis. Clin. North Am. 31, 731–742. 10.1016/j.idc.2017.07.010 29079157

[B30] Rudd K. E. Johnson S. C. Agesa K. M. Shackelford K. A. Tsoi D. Kievlan D. R. (2020). Global, regional, and national sepsis incidence and mortality, 1990–2017: analysis for the Global Burden of Disease Study. Lancet 395, 200–211. 10.1016/S0140-6736(19)32989-7 31954465 PMC6970225

[B43] Schmitt C. A. Tchkonia T. Niedernhofer L. J. Robbins P. D. Kirkland J. L. Lee S. (2023). COVID-19 and cellular senescence. Nat. Rev. Immunol. 23, 251–263. 36198912 10.1038/s41577-022-00785-2 PMC9533263

[B31] Schulz L. Hornung F. Häder A. Radosa L. Brakhage A. A. Löffler B. (2024). Influenza virus-induced paracrine cellular senescence of the lung contributes to enhanced viral load. Aging Dis. 14, 1331–1348. 10.14336/AD.2023.0310 PMC10389813 37163429

[B32] Singer M. Deutschman C. S. Seymour C. W. Shankar-Hari M. Annane D. Bauer M. (2016). The third international consensus definitions for sepsis and septic shock (sepsis-3). JAMA 315, 801–810. 10.1001/jama.2016.0287 26903338 PMC4968574

[B33] Trevisan C. Noale M. Amidei C. B. Ferroni E. Basso C. Fedeli U. (2023). Frailty and the risk of infection-related hospitalizations in older age: differences by sex. Maturitas 168, 1–6. 10.1016/j.maturitas.2022.10.009 36370488

[B34] van den Berg M. van Beuningen F. E. ter Maaten J. C. Bouma H. R. (2022). Hospital-related costs of sepsis around the world: a systematic review exploring the economic burden of sepsis. J. Crit. Care 71, 154096. 10.1016/j.jcrc.2022.154096 35839604

[B35] Victorelli S. Passos J. F. (2017). Telomeres and cell senescence - size matters not. EBioMedicine 21, 14–20. 10.1016/j.ebiom.2017.03.027 28347656 PMC5514392

[B36] Wang S. Yao X. Ma S. Ping Y. Fan Y. Sun S. (2021). A single-cell transcriptomic landscape of the lungs of patients with COVID-19. Nat. Cell Biol. 23, 1314–1328. 10.1038/s41556-021-00796-6 34876692 PMC8650955

[B37] Wang Z. Ma Y. Chen Z. Yang R. Liu Q. Pan J. (2023). COVID-19 inhibits spermatogenesis in the testes by inducing cellular senescence. Front. Genet. 13, 981471. 10.3389/fgene.2022.981471 36685935 PMC9849386

[B45] Wrona M. V. Ghosh R. Coll K. Chun C. Yousefzadeh M. J. (2024). The 3 I’s of immunity and aging: immunosenescence, inflammaging, and immune resilience. Front. Aging 5. 10.3389/fragi.2024.1490302 PMC11521913 39478807

[B42] Yoshikawa T. T. Norman D. C. (2017). Geriatric infectious diseases: Current concepts on diagnosis and management. J. Am. Geriatr. Soc. 65, 631–641. 28140454 10.1111/jgs.14731

[B38] Zhang L. Pitcher L. E. Prahalad V. Niedernhofer L. J. Robbins P. D. (2023). Targeting cellular senescence with senotherapeutics: senolytics and senomorphics. FEBS J. 290, 1362–1383. 10.1111/febs.16350 35015337

[B39] Zheng Y. Liu X. Le W. Xie L. Li H. Wen W. (2020). A human circulating immune cell landscape in aging and COVID-19. Protein Cell 11, 740–770. 10.1007/s13238-020-00762-2 32780218 PMC7417788

