# Supplementary material for: Cellular senescence in acute human infectious disease: a systematic review
Source: Front Aging. 2024 Nov 15;5:1500741. doi: 10.3389/fragi.2024.1500741 (PMC11604623; doi:10.3389/fragi.2024.1500741)
Supplement: Supplementary file 2 [file Table1.docx]

**Table 1.** Summary of studies included in the analysis with their infectious disease of interest, number of subjects and controls, and measured senescence markers.

| **Study** | **Pathogen** | **Participants (n)** | **Healthy Controls (n)** | **Senescence Markers** |
| --- | --- | --- | --- | --- |
| Evangelou et. al. 2022^†‡^ | SARS-CoV-2 | 11 | 43 | p16^INK4a^, ɣH2AX, SASP, SenTraGor™ |
| Froidure et. al. 2020^†^ | SARS-CoV-2 | 70 | 491 | SA-β-gal, Telomere length |
| Lee et. al. 2021^†^ | SARS-CoV-2 | 24 | 5 | p16^INK4a^, p21^CIP1^, H3K9me3, SASP, Lipofuscin |
| Lekva et. al. 2022^†‡^ | SARS-CoV-2 | 97 | 22 | p16^INK4a^, p21^CIP1^, SA-β-gal, Telomerase activity |
| Lin et. al. 2023^†‡^ | SARS-CoV-2 | 24 | 12 | p16^INK4a^, p21^CIP1^, SASP |
| Lipskaia et. al. 2022^†‡^ | SARS-CoV-2 | 9 | 2 | p16^INK4a^, p21^CIP1^, SASP |
| Nguyen et. al. 2022^†‡^ | SARS-CoV-2 | 28 | 8 | SASP |
| Evangelou et. al. 2021^‡^ | SARS-CoV-2 | 10 | 10 | P16, SASP, SenTraGor™ |
| Roh et. al. 2022^†‡^ | SARS-CoV-2 | 54 | 26 | SASP |
| Wang et. al., 2021^‡^ | SARS-CoV-2 | 5 | 4 | p16^INK4a^, p21^CIP1^, p53, SASP |
| Wang et. al., 2023^†^ | SARS-CoV-2 | 3 | 3 | mTOR, MAPK, p53 pathways |
| Zheng et. al. 2020^†‡^ | SARS-CoV-2 | Cohort 1: 56* Cohort 2: 8 Cohort 3: 22 |  | SASP |
| Fantecelle et. al. 2021^†^ | *Leishmania Braziliensis* | 21 | 7 | p16^INK4a^, p21^CIP1^, p38, ATM, SASP |
| Covre et. al. 2019^†‡^ | *Leishmania Braziliensis* | 17 | 15 | ɣH2AX, SASP, Telomere length |
| Asghar et. al. 2018^†‡^ | *Plasmodium Falciparum* | 38 | 38 | p16^INK4a^, Telomere length, Telomerase activity |
| Martin-Escolano et. al. 2023^†‡^ | Hepatitis C | 32 | 24 | SASP, Immune checkpoint biomarkers |

*Cohort 1 consisted of young healthy adults (20-45 years old) and aged healthy adults (≥60 years old); Cohort 2 consisted of young health adults (30-45 years old), aged healthy adults (≥60 years old), young SARS-CoV-2 (30-50 years old), and aged SARS-CoV-2 (≥70 years old); Cohort 3 consisted of young health adults (30-45 years old), aged healthy adults (≥60 years old), young recovered SARS-CoV-2 (30-50 years old), and aged recovered SARS-CoV-2 (≥70 years old).

†Denotes studies clarifying gender distribution.

‡ Deontes studies clarifying age-matched controls.

Abbreviations: p16^INK4a^ (cyclin dependent kinase inhibitor 2A); p21^CIP1^ (cyclin dependent kinase inhibitor 1); SASP (Senescence associated secretory phenotype); SenTraGor™ (Antibody enhanced detection of senescent cells); yH2AX (gamma H2A histone family member X), SA-β−gal (Senescence associated beta-galactosidase activity); H3K9me3 (Histone H3 Lysine 9 trimethylation); GDF15 (Growth differentiation factor 15); F3 (Coagulation factor III); mTOR (mammalian target of rapamycin); MAPK (mitogen activated protein kinase); p53 (Tumor protein P53); p38 (mitogen activated protein kinase 14); ATM (ataxia telangiectasia mutated)
