# Supplementary material for: Cellular senescence in acute human infectious disease: a systematic review
Source: Front Aging. 2024 Nov 15;5:1500741. doi: 10.3389/fragi.2024.1500741 (PMC11604623; doi:10.3389/fragi.2024.1500741)
Supplement: Supplementary file 3 [file Table2.docx]

**Table 2.** Summary of findings from studies included in the analysis specifically investigating SARS-CoV-2.

| **Study** | **Senescence Markers** | **Sample type** | **Primary findings** |
| --- | --- | --- | --- |
| Evangelou et. al. 2022 | p16, yH2AX, SASP, SenTraGor | AT2 Lung Cells | ↑p16, ↑SenTraGor positivity, and ↑SASP (*p* < 0.0001) |
| Froidure et. al. 2020 | SA-β−gal, Telomere length | Leukocytes | Shorter telomeres than controls (*p* < 0.001) and ↑SA- β−gal positivity |
| Lee et. al. 2021 | p16, p21, H3K9me3, SASP, Lipofuscin | Airway Mucosa; macrophages | ↑p16, ↑p21, ↑H3K9me3, ↑ lipofuscin, and ↑IL8 |
| Lekva et. al. 2022 | p16, p21, SA- β−gal, telomere-associated SASP | Plasma | ↑p16 and telomere-associated SASP but normal p21 and SA- β−gal 3 months after hospitalization (*p* < 0.05). |
| Lin et. al. 2023 | p16, p21, SASP | PBMC | p21 (*p* < 0.05) correlated with disease severity and SenMayo SASP gene-set was upregulated |
| Lipskaia et. al. 2022 | p16, p21, SASP, GDF15 | Epithelial ciliated; club cells | ↑p16, ↑p21, ↑uPAR, ↑CXCL8, ↑IGFBP3, and ↑GDF15 (*p* < 0.0001) |
| Nguyen et. al. 2022 | SASP, F3 | Macrophages; Epithelial cells | Macrophages had moderate levels of SASP. Epithelial cells displayed ↑SASP in SARS-CoV-2 patients with increased expression of F3 in severe cases (*p* < 0.05) |
| Evangelou et. al. 2021 | p16, SASP, SenTraGor | AT2 Lung Cells | Greater reactivity to SenTraGor, ↑p16 immunostaining, and co-expression of IL-1β and IL-6. |
| Roh et. al. 2022 | SASP | Plasma | ↑SASP expression with cardiac involvement of SARS-CoV-2 (*p* < 0.05). |
| Wang et. al., 2021 | p16, p21, p53, SASP | Lung Tissue | ↑p16, ↑p21, ↑IL-6, ↑p53, and ↑SASP |
| Wang et. al., 2023 | mTOR, MAPK, p53 pathways | Testicular Tissue | Senescence mediated by MAPK (*r* = 0.999, *p* < 0.001), mTOR, and p53 signaling is positively correlated with SARS-CoV-2 disease in testes. |
| Zheng et. al. 2020 | SASP | Peripheral T-Cells; monocytes | ↑SASP hallmark genes (*CDKN* family) |
