# Supplementary material for: Cellular senescence in acute human infectious disease: a systematic review
Source: Front Aging. 2024 Nov 15;5:1500741. doi: 10.3389/fragi.2024.1500741 (PMC11604623; doi:10.3389/fragi.2024.1500741)
Supplement: Supplementary file 4 [file Table3.docx]

**Table 3.** Summary of findings from studies included in the analysis investigating *Leishmania braziliensis*, *Plasmodium falciparum*, or hepatitis C.

| **Study** | **Disease** | **Senescence Markers** | **Sample Type** | **Primary Findings** |
| --- | --- | --- | --- | --- |
| Asghar et. al. 2018 | *P. falciparum* | p16, Telomere length, Telomerase activity | Whole Blood | ↑p16, ↓telomerase activity, and telomere shortening (*p* < 0.05). Over time telomerase activity increases and telomere length is gradually restored. |
| Covre et. al. 2018 | *L. braziliensis* | γH2AX, Telomere length, Telomerase activity, CD57 | CD8 T Cells | ↑expression of SASP markers including CD57, KLRG1, p38, and γH2AX (*p* < 0.001), ↓telomeres (*p* < 0.0001) and ↓telomerase expression (*p* < 0.001). |
| Fantecelle et. al. 2021 | *L. braziliensis* | p16, p21, p38, ATM, Sestrin 2 | Skin | ↑p16, p21, p38, ATM, and Sestrin 2 (*p* < 0.0001); positively correlated with lesion size and parasitic load and independent of patient age. |
| Martin-Escolano et. al. 2023 | Hepatitis C | SASP, immune checkpoint biomarkers | Plasma | ↑SASP and immune checkpoint biomarkers more than 2 years after infection (*p* < 0.05). |
