# Supplementary material for: Cellular senescence in acute human infectious disease: a systematic review
Source: Front Aging. 2024 Nov 15;5:1500741. doi: 10.3389/fragi.2024.1500741 (PMC11604623; doi:10.3389/fragi.2024.1500741)
Supplement: Supplementary file 5 [file DataSheet1.docx]

Cellular senescence and infection - Search Strategy

Will Miller

Updated 8-8-2022

### Databases:

1. Ovid Medline - 5064
2. Embase - 7532
3. Scopus - 7063
4. Web of Science - 7243
5. Cochrane - 186
6. ClinicalTrials.gov - 573
7. Global Index Medicus - 230

**Searches executed:** 08/08/2022

**Total before duplicates removed:** 27,920

**Total after duplicates removed:** 13,579

**Total in Covidence after duplicates removed:** [#]

###

### Ovid Medline (5064)

| **#** | **Search Statement** | **Results** |
| --- | --- | --- |
| 1 | exp cellular senescence/ | 25570 |
| 2 | ((cell* and senescen*) or immunosenescen*).ti,ab,kw,kf. | 35886 |
| 3 | 1 or 2 | 48154 |
| 4 | exp infections/ | 2967076 |
| 5 | (infect* or (acute adj2 phase) or T-cell* or B-cell*).ti,ab,kw,kf. | 2515071 |
| 6 | 4 or 5 | 4226485 |
| 7 | 3 and 6 | 6086 |
| 8 | exp animals/ | 25707257 |
| 9 | exp humans/ | 20672184 |
| 10 | 8 not 9 | 5035073 |
| 11 | 7 not 10 | 5064 |

###

### Embase (7532)

| **#** | **Search Statement** | **Results** |
| --- | --- | --- |
| 1 | exp cell aging/ | 27201 |
| 2 | ((cell* and senescen*) or immunosenescen*).ti,ab,kw,kf. | 47633 |
| 3 | 1 or 2 | 58922 |
| 4 | exp infection/ | 4283505 |
| 5 | (infect* or (acute adj2 phase) or T-cell* or B-cell*).ti,ab,kw,kf. | 3369734 |
| 6 | 4 or 5 | 5658131 |
| 7 | 3 and 6 | 8981 |
| 8 | exp animal/ | 31032180 |
| 9 | exp human/ | 25264165 |
| 10 | 8 not 9 | 5768015 |
| 11 | 7 not 10 | 7532 |

###

### Scopus (7063)

( ( ( INDEXTERMS ( "cellular senescence" OR "cell aging" ) ) OR ( TITLE-ABS-KEY ( ( cell* AND senescen* ) OR immunosenescen* ) ) ) AND ( ( INDEXTERMS ( infection OR infections ) ) OR ( TITLE-ABS-KEY ( infect* OR ( acute W/2 phase ) OR t-cell* OR b-cell* ) ) ) ) AND NOT ( INDEXTERMS ( animals OR animal ) AND NOT ( INDEXTERMS ( humans OR human ) ) )

### ClinicalTrials.gov (573)

**Condition or Disease:** infections

**Other terms:** cellular senescence

### Global Index Medicus (230)

(tw:((mh:(“cellular senescence”) or (tw:((cell* and senescen*) or immunosenescen*)) AND (mh:(“infections” or “infection”) OR (tw:(infect* or (acute phase) or T-cell* or B-cell*)))))

### Web of Science (7242)

Indexes=SCI-EXPANDED, SSCI, A&HCI, CPCI-S, CPCI-SSH, BKCI-S, BKCI-SSH, ESCI, CCR-EXPANDED, IC Timespan=All years

| **#** | **Results** | **Search Statement** |
| --- | --- | --- |
| # 1 | 7242 | TS=((cell* and senescen*) or immunosenescen*) AND TS=(infect* or (acute NEAR/2 phase) or T-cell* or B-cell*) |

###

### Cochrane (186)

| **ID** | **Search** | **Hits** |
| --- | --- | --- |
| #1 | MeSH descriptor: [Cellular Senescence] explode all trees | 84 |
| #2 | ((cell* and senescen*) or immunosenescen*) | 456 |
| #3 | #1 or #2 | 500 |
| #4 | MeSH descriptor: [Infections] explode all trees | 82244 |
| #5 | (infect* or (acute NEAR/2 phase) or T-cell* or B-cell*) | 176972 |
| #6 | #4 or #5 | 202809 |
| #7 | #3 and #6 | 186 |
